# Spotlight on early-career researchers: an interview with Kate Miroshnikova

**DOI:** 10.1038/s42003-019-0450-2

**Published:** 2019-05-21

**Keywords:** Careers, Lab life, Barrett oesophagus, Cell biology

## Abstract

Dr. Kate Miroshnikova is a bioengineer by training and is currently a postdoctoral EMBO/HFSP fellow at the Max Planck Institute for Biology of Ageing and at the Helsinki Institute of Life Science. She is interested in understanding biomechanical regulation of stem cell fate decisions in health and disease. Kate’s long term scientific interest is to understand how cells and tissues sense, integrate, and adapt their transcriptomes and proteomes to the highly dynamic mechanical environments without compromising structural and genomic integrity.

**Figure:**
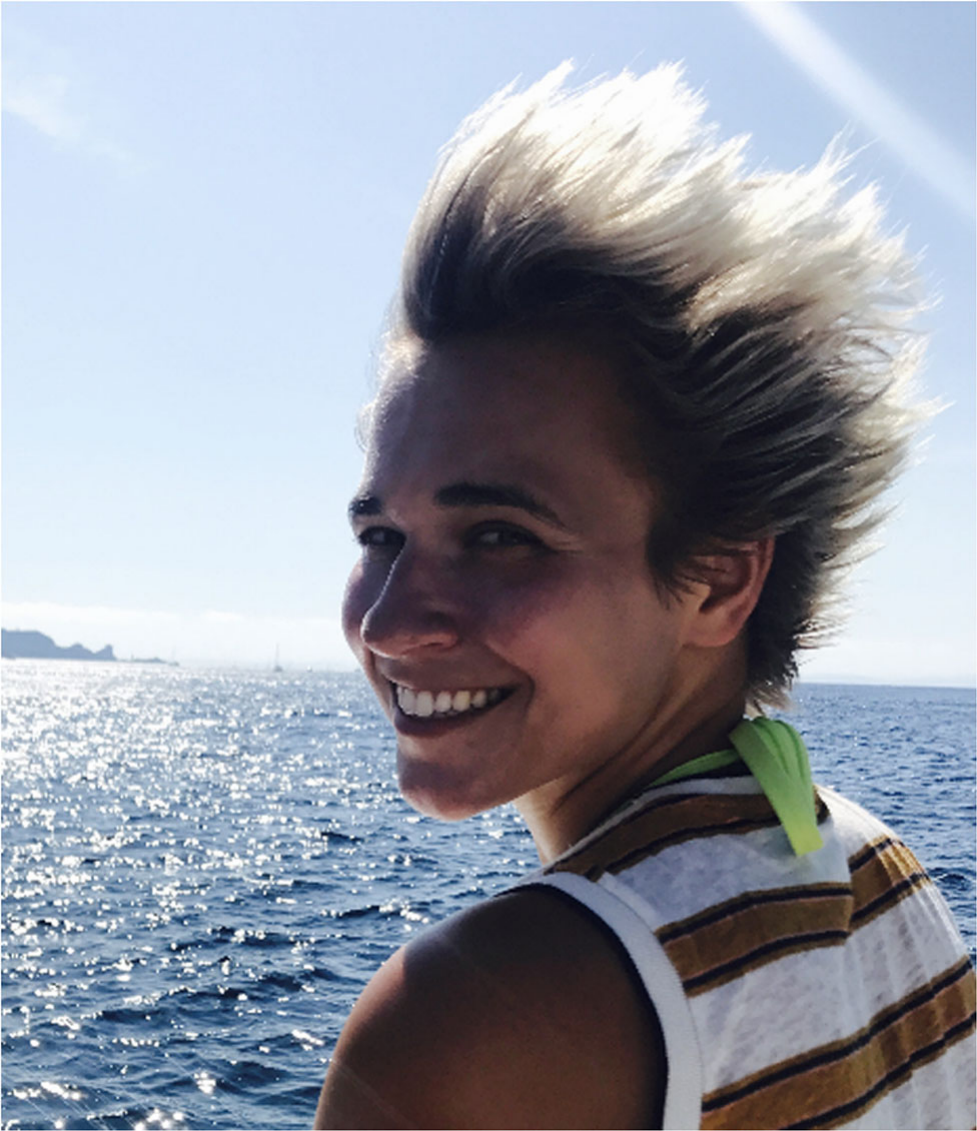
Image credit: Kate Miroshnikova

Please tell us about your research interests

I am passionate about understanding how biophysical and mechanical cues affect cell and tissue behaviour. Physical forces act on our tissues in a dynamic manner at all times—from the pulsatile blood flow, shear stress and cyclic strain in the vasculature to the continuous stretch and compression of the skin and the constant contraction of the heart, which keeps us alive. All this occurs under normal physiological conditions. But we also now know that forces, and how cells respond to them, are altered in many diseases, especially cancer. Although we have known about the existence of these physical stimuli for quite a while, only very recent technological developments, particularly in imaging, have allowed us to precisely quantify mechanical aspects of biology and thus to manipulate them. So it is a great time to be a mechanobiologist. May the force be with us!

It is easy to comprehend the compression of brain tissue in the case of pathological fluid accumulation within the confines of a rigid skull or the hardening of cancerous breast tissue that manifests as palpable lumps. We now know that these changes in tissue biophysics are sensed by cells and transduced into meaningful biological events via a process known as mechanotransduction. My scientific journey began as a PhD student with quantifying the extracellular biophysical changes in breast, pancreatic, and brain cancers and figuring out relevant, often druggable, pathways affected by these changes. During my postdoctoral tenure in Grenoble, France, I focused on understanding the molecular underpinnings of force-sensing machineries at the cellular periphery in the context of cardiovascular diseases. More recently, I became interested in deciphering the physiological roles of biophysical forces, especially as it applies to specifying fate choices in epidermal stem cells. We discovered that changes in local mechanics drive stem cell differentiation while at the same time allowing proper stem cell positioning within the tissue. Currently I am expanding on this work to understand how mechanical signals are actually propagated into the nucleus to regulate cell fate. I am particularly excited about the impact of forces on chromatin that lies directly in the path of force transduction. What we are finding now is that mechanical cues from the extracellular environment are not only directly sensed by the chromatin but they also impact chromatin organization and modify its biophysical properties. Given this, I am currently excited to uncover the mechanoprotective mechanisms that maintain genomic integrity despite constant mechanical insults in both health and disease.

Why did you choose to be a scientist?

I am not sure I would have been able to formalize it at the onset of my journey as a scientist/bioengineer, but freedom of thought, the sheer lack of existing bounds of knowledge and creativity is why I wanted to become a scientist. It is also why I want to remain a part of the scientific community. Every day I think of why certain phenomena occur and I design experiments to test my informed guesses at why life is as it is and the only limit is my own imagination. In this sense those of us who are a part of the scientific process are truly privileged.

What are your predictions for your field in the near future?

I expect great things from physicists, mathematicians, and engineers coming together with biologists and clinicians to solve problems that are relevant to human health. I predict that, thanks to this interdisciplinary effort, we will be doing experiments in silico to generate more precise hypotheses to be tested in the wet lab. We will be characterizing the mechano-phenotype of many diseases. In fact, we are already diagnosing cancer tissues based on differential mechanics without formally defining it as such. I think that there are many avenues for disease onset—some are purely genetic/biochemical, some are mechanical, and most are probably a combination of the two—and it is only rational to consider tissue mechanics-normalizing therapeutic strategies going forward.

Can you speak of any challenges that you have overcome?

I feel quite lucky to have had tremendous support and mentorship, in addition to doing visionary science, from advisors starting from my undergraduate studies all the way to my current postdoctoral tenure. I have faced many professional challenges, especially related to the occasionally overwhelming workload, time management issues, and struggle to meet deadlines, but I would not place these out of the ordinary for a stimulating job. Having said that, in the past 4 years I have lived in 4 different countries and while that has been exciting at times, it has taken me away from my family and it’s sometimes difficult for me to say where my home is anymore. This is a common challenge for many scientists, as we need to go where science takes us.

What advice would you give to your younger self?

You always see more clearly in hindsight and perhaps that is the best part of riding the rollercoaster that is academic research. I made many mistakes but I also learned my best lessons while making them. These mistakes made me a better researcher and colleague so I would make them all over again. I would tell myself to trust my own instincts more when making big decisions. I would also tell myself to invest even more time establishing and nourishing a network of peers, mentors and colleagues. Science is a team sport!

What is the funniest or most memorable thing that has happened to you in the lab?

What matters the most to me as a person is the many professional relationships that a career in academia has allowed me to build. Science can be quite lonely and discouraging at times, for instance when struggling to make experiments work and dealing with rejections but my overall experience has been overwhelmingly positive thanks to great colleagues. Some experiences that come to mind are the countless laughs at the atomic force microscope with a Moroccan colleague during my PhD, many impromptu dance moves during my short postdoctoral tenure in France with a colleague from Bulgaria, and these days, daily singalongs to Whitney Houston and Tina Turner with a Croatian colleague in the lab. These moments matter every day.

*This interview was conducted by Senior Editor Christina Karlsson-Rosenthal*.

